# Modified Harrington’s procedure for periacetabular metastases in 89 cases: a reliable method for cancer patients with good functional outcome, especially with long expected survival

**DOI:** 10.1080/17453674.2020.1732016

**Published:** 2020-02-28

**Authors:** Gilber Kask, Jyrki Nieminen, Vincent van Iterson, Mihhail Naboistsikov, Toni-Karri Pakarinen, Minna K Laitinen

**Affiliations:** a Department of Orthopaedics and Traumatology, Tampere University Hospital, Tampere;; b Coxa Hospital for Joint Replacement, Tampere;; c Department of Orthopaedics and Traumatology, Helsinki University Hospital, University of Helsinki, Helsinki, Finland

## Abstract

Background and purpose — The pelvis is the 3rd most common site of skeletal metastases. In some cases, periacetabular lesions require palliative surgical management. We investigated functional outcome, complications, and implant and patient survival after a modified Harrington’s procedure.

Patients and methods — This retrospective cohort study included 89 cases of surgically treated periacetabular metastases. All patients were treated with the modified Harrington’s procedure including a restoration ring. Lesions were classified according to Harrington. Functional outcome was assessed by Harris Hip Score (HHS) and Oxford Hip Score (OHS). Postoperative complications, and implant and patient survival are reported.

Results — The overall postoperative functional outcome was good to fair (OHS 37 and HHS 76). Sex, age, survival > 6 and 12 months, and diagnosis of the primary tumor affected functional outcome. Overall implant survival was 96% (95% Cl 88–100) at 1 year, 2 years, and 5 years; only 1 acetabular implant required revision. Median patient survival was 8 months (0–125). 10/89 patients had postoperative complications: 6 major complications, leading to revision surgery, and 4 minor complications.

Interpretation — Our modified Harrington’s procedure with a restoration ring to achieve stable fixation, constrained acetabular cup to prevent dislocation, and antegrade iliac screws to prevent cranial protrusion is a reliable reconstruction for periacetabular metastases and results in a good functional outcome in patients with prolonged survival. A standardized procedure and low complication rate encourage the use of this method for all Harrington class defects.

The pelvis is the 3rd most common site for surgically treated skeletal metastases after the femur and humerus (Ratasvuori et al. 2013).

In deciding whether and how to operate on periacetabular lesions, the estimated patient survival and size of the skeletal lesion should be considered. Expected survival is dependent on the type of primary tumor and metastatic burden. The mean survival of pathological fractures in the pelvic area is usually less than 2 years (Hansen et al. 2004, Ratasvuori et al. 2014).

Periacetabular defects can be reconstructed in several ways depending on the extent. Harrington’s classification separates cases as follows: class I, the acetabular lateral cortices and superior and medial walls are intact; class II, the medial wall is deficient; class III, the lateral cortices, medial wall, and superior wall are all deficient; and class IV, there is wide destruction all the way to the wing of the ilium (Harrington 1981). Harrington also designed a method for reconstruction in cases in which the periacetabular bone presents extensive loss, as in classes III and IV. In this conventional procedure, antegrade pins (from the wing of the ilium to the acetabular dome) or retrograde pins (from the acetabular dome into the wing of the ilium and into the sacroiliac joint) are used. Other methods are also available for the reconstruction, such as filling metastatic cavities with bone cement (cementoplasty), acetabular cages, custom-made pelvic endoprostheses, and the “ice-cream cone” periacetabular prosthesis (Walker 1993, Harrington 1995, Fisher et al. 2011).

The original Harrington’s procedure is rarely used any more, whereas some studies have other procedures, usually less invasive, e.g., no pins in the iliac crest (Tsagozis et al. 2015), using short screws or pins (Bernthal et al. 2015, Tsagozis et al. 2015), and not performing arthroplasty (Charles et al. 2017). Sample sizes in publications reporting conventional and modified Harrington’s technique are small, ranging from 19 to 51 patients (Harrington 1981, Nilsson et al. 2000, Tillman et al. 2008, Shahid et al. 2014, Charles et al. 2017), and the publications reporting functional outcomes are few (Nilsson et al. 2000, Charles et al. 2017).

The aim of this study was to report the functional outcome, post-operative implant survival, including complications, and patient survival after modified Harrington’s procedure.

## Patients and methods

### Study design

The medical records from prospectively maintained hospital databases were reviewed retrospectively. 89 periacetabular metastasis cases treated surgically with the modified Harrington’s procedure at Helsinki University Hospital, Helsinki, and Coxa Replacement Hospital, Tampere, Finland, were included in the analysis. Patients were included if they met the following inclusion criteria: age ≥ 18 years, diagnosis of metastatic disease in the pelvic periacetabular bone, and surgery for an impending fracture or existent pathological fracture of the periacetabular area between January 2006 and December 2018 (Table 1). Patients with (multiple) myeloma and lymphoma were also included because the surgical approach for these diseases is similar to the treatment of metastatic long bone disease. No predefined criteria were used by the surgeons to make the decision to operate; the patient and physician together decided whether to operate.

**Table 1.** Patient characteristics. Values are number of cases unless otherwise specified

Characteristics	n
Eligible cases	89
Sex	
Female	50
Male	39
Mean/median follow-up months (range)	18/8 (0–125)
Mean age at surgery years (range)	67 (27–94)
Age	
	62
≤ 60	27
Metastatic load	
Multiple bone	43
Solitary bone	18
Bone, lung, and other	17
Bone and lung	11
ASA	
2	14
3	41
4	26
Data missing	8
Radiotherapy	
Preoperative	31
Postoperative	37
Pre- and postoperative	2
None	19
Primary malignant tumor	
Breast	28
Prostate	13
Renal cell	11
Myeloma	10
Lung	7
Other	20
Last status	
Dead due to cancer	55
Dead due to treatment	3
Dead due to other cause	1
Dead due to unknown reason	10
Alive	20

Preoperative radiological assessment was performed by plain radiographs, CT, and/or MRI. Metastatic lesions were classified according to Harrington (Harrington 1981).

### Surgical procedure

All procedures were performed by orthopedic oncologists, and a similar procedure was performed on all patients independent of Harrington’s classification. Patients were placed under spinal anesthesia, and a preoperative prophylactic antibiotic was used. In our modified Harrington’s technique, the procedure was started by a posterior approach. The hip was dislocated and the neck resected. Curettage of periacetabular metastases was performed, and then cannulated mostly fully threaded, occasionally partially threaded 7.3 mm screws were inserted from the iliac crest through a separate transverse iliac incision. The screws were directed to the roof of the acetabulum. The periacetabular defect was then supported by adding a Restoration GAP II reinforcement ring (Stryker, Mahwah, NJ, USA). A conventional normal or constrained cemented acetabular cup was implanted, followed by the femoral component, and bone cement was used to augment the bone defects, reinforcement ring, and antegrade inserted screws (Figure). Various components were used in both the femur and acetabulum throughout the study period (Table 2). Preoperative embolization was selectively performed in 15 cases. The majority of patients received postoperative radiation therapy. All patients were mobilized postoperatively, allowing immediate full weight-bearing. Postoperative clinical assessment was performed routinely after 2–3 months, 6 months, and 12 months.

**Table 2.** Perioperative characteristics of the 89 patients. Values are number of cases unless otherwise specified

Characteristics	n
Operation	
Primary only	83
Revision	6
Mean lesion size (cm) (range)	8.9 (4–25)
Harrington’s classification	
Class I	36
Class II	41
Class III	12
Class IV	–
Preoperative embolization	
Yes	15
No	69
Data missing	5
Mean operation blood loss (L) (range)	1.6 (0.1–5.7)
Impending fracture	33
Pathologic fracture	56
Mean operation time (hr) (range)	2.8 (2.2–7.2)
Type of prothesis	
Cemented regular	70
Uncemented regular	2
Cemented long-stem	7
Uncemented long-stem	3
Tumor prothesis	7
Acetabulum component	
Constrained	56
Normal	33
Antegrade acetabular pins (n)	
2	3
3	68
4	11
5	7
Head size (mm)	
22	2
28	11
30	1
32	18
36	57

### Functional outcome

Functional outcome was assessed by the Harris Hip Score (HHS) and the Oxford Hip Score (OHS). To report the level of the functional outcome, we used the grading system by Marchetti et al. (2005). Functional outcomes based on HHS were: < 70, poor; 70–79, fair; 80–89, good; and 90–100, excellent.

The OHS is a patient-reported outcome measure (PROM) devised as a joint-specific instrument. A score > 41 is considered excellent, 34–41 good, 27–33 fair, and < 27 poor functional outcome (Kalairajah et al. 2005). To report a good functional outcome, we used the definition by Hamilton et al. (2018) of “treatment success” for THA patients based on an OHS threshold of 37.5 points.

Questionnaires were not used for patients with < 2 months of postoperative follow-up or in bad general health, or when patients were confined to bed or disoriented by advanced disease.

### Complications

Minor, major, and mechanical postoperative complications are reported. Complications were deemed major when surgical reintervention was needed. Mechanical complications were defined as an implant failure. Implant survival was defined as the time from the Harrington procedure to revision due to any cause.

### Statistics

Statistical analyses were performed using SPSS Statistics 23.0 (IBM Corp, Armonk, NY, USA). A p-value < 0.05 was considered significant. The Kaplan–Meier method was applied for patient and implant survival. Patient survival rates were calculated from the date of surgery to the most recent follow-up or death, and implant survival to revision surgery due to a failure of the reconstruction. Between-group comparisons were performed using the log-rank test. Continuous variables are reported as means and 95% confidence intervals (CI). The chi-squared test or Fisher’s exact test was used to compare variables between groups, and the Kruskal–Wallis test for means between groups. Linear regression analysis was performed to determine the relation in scores in a time-series analysis.

### Ethics, funding, data sharing plan, and potential conflicts of interest

This retrospective cohort study was approved by the local chair of the audit department. This research received no specific grant or funding from any funding agency in the public, commercial, or not-for-profit sectors. The data are available from the corresponding author. No competing interests are declared.

## Results

### Functional outcome

Functional outcome data were collected from 53/89 patients. The OHS was determined for 18/89 patients and the HHS for 51/89. Data were missing for 36/89 patients: 14/89 survived < 2 months, and 22/89 could not answer the questions due to bad general health. Functional outcome measurements were performed on average at 1 year (OHS at 17 months and HHS at 9 months).

The average postoperative OHS was 37 (good), and 6/18 of the patients had successful treatment (> 37.5 points) according to Hamilton’s definition (Hamilton et al. 2018). The average HHS was 76, but was statistically significantly better in female patients (82 versus 69; p = 0.01), patients aged < 60 years (87 versus 71; p = 0.001), and in patients with survival > 6 months (79 versus 67; p = 0.03) and > 12 months (81 versus 68; p = 0.01). The HHS was better in patients with prostate cancer (85; p = 0.04) and worse in patients with myeloma (55; p = 0.04) (Table 3). From linear regression analysis, we could not detect a statistically significant increase in scores and time.

**Table 3.** Functional outcome scores

	OHS score			HHS score		
Characteristics	n	mean	p-value	n	mean	p-value
Eligible cases	18	37		51	76	
Sex			0.6			0.01
Female	8	38		26	82	
Male	10	36		25	69	
Age over 60 years			0.5			0.001
Yes	15	36		36	71	
No	3	41		15	87	
Metastatic load			1.0			1.0
Multiple bone	10	37		27	76	
Solitary bone	6	37		10	76	
Bone and other	2	37		14	76	
ASA			0.2			0.1
2	3	46		9	86	
3	5	32		24	74	
4	4	38		12	70	
Radiotherapy			0.5			0.8
Preoperative	5	34		16	76	
Postoperative	8	40		27	76	
Pre- and post.	0	–		1	94	
None	5	36		7	73	
Harrington’s classification			0.9			0.3
Class I	4	36		17	79	
Class II	10	37		24	72	
Class III	4	38		10	80	
Primary malignant tumor			1.0			0.04
Breast	6	37		19	85	
Prostate	3	40		9	72	
Renal cell	2	33		6	55	
Myeloma	3	35		8	75	
Lung	6	37		19	85	
Other	1	43		4	81	
Survival over 6 months			0.4			0.03
Yes	16	36		38	79	
No	2	42		13	67	
Survival over 12 months			0.7			0.01
Yes	15	37		30	81	
No	3	35		21	68	
Operation						0.7
Primary only	18	37		48	75	
Revision	–	–		3	80	
Acetabulum component			0.3			0.8
Constrained	14	36		38	76	
Normal	4	41		13	75	

### Implant survival and complications

Overall implant survival was 96% (Cl 88–100) at 1 year, 2 years, and 5 years. Harrington’s classification (p = 0.9), radiotherapy (p = 0.3), sex (p = 0.3), and type of primary tumor (p = 0.3) did not statistically significantly influence implant survival at univariable analysis.

10/89 postoperative complications occurred: 6 major, leading to revision surgery, and 4 minor, which were treated nonoperatively. 1 patient had mechanical failure of the initial construct due to constant dislocation, necessitating re-intervention at 6 months. The postoperative infection rate was 4/89 (Table 4). We observed fewer dislocations when using constrained cups (1/56 patient) when compared with normal cups (3/33 patients) (p = 0.04).

**Table 4.** Complications of the Harrington’s procedure cases

Complications	Mechanical failure	Hip joint dislocation	Local tumor progression	Infection	Decubitus
Major	1	2	0	2	1
Minor	0	2	1	2	0
Overall	1	4	1	4	1

### Patient survival

55/89 of the patients died of cancer. 3 died due to treatment: 1 due to fatal pulmonary embolism and 2 due to consequences of deep infection and sepsis. Overall patient survival was 46% (Cl 35–57) at 1 year, 25% (Cl 14–35) at 2 years, and 16% (Cl 7–25) at 5 years. Median patient survival was 8 months (0–125). In patients with skeletal and lung metastases, the overall survival was 50% at 4 months and 23% at 1 year. The survival was significantly better in patients with skeletal metastases only, compared with patients with more disseminated disease (p < 0.001). We could not observe a statistically significant difference in survival between patients with solitary or multiple skeletal metastases, nor did we detect a difference between different Harrington’s classifications on patient survival.

## Discussion

We used our modified Harrington technique, namely the insertion of antegrade screws from the iliac crest and an antiprotrusion cup combined with THA, in all patients with periacetabular metastasis and pathological fractures regardless of the size of the lesion. Our results show that the reconstruction is durable, with low implant-related failure and complication rates, and good functional outcomes.

Functional outcomes have been reported in a few studies, usually in publications with small sample sizes of Harrington or modified Harrington procedure (range 19 to 51 patients) (Harrington 1992, Nilsson et al. 2000, Tillman et al 2008, Shahid et al. 2014, Charles et al. 2017), or with non-specific outcome measures (Tillman et al. 2008, Charles et al 2017, Wegrzyn et al. 2018, Rowell et al. 2019). No studies have been published using PROMs. Most of the studies have used ClinROMs, such as the Musculoskeletal Tumor Society (MSTS) score (Harrington 1992, Allan et al. 1995) or the HHS (Wegrzyn et al. 2018), which was developed for the assessment of hip surgery outcomes and is intended to evaluate various hip disabilities and methods of treatment in an adult population. In accordance with the literature, our results shows that about half of periacetabular metastasis patients could ambulate in the community independently after such large reconstructive surgery (Wegrzyn et al. 2018, Rowell et al. 2019).

The OHS is a validated, reliable, and well-established PROM for evaluating the outcomes of THA (Dawson et al. 1996). It is difficult to compare our results with the literature, as postoperative mobility has been reported in different ways (Harrington 1981, Marco et al. 2000, Shahid et al. 2014, Charles et al. 2017, Wegrzyn et al. 2018, Rowell et al. 2019). Based on Marchetti’s grading system (Marchetti et al. 2005), the functional outcome results were fair in our population when all patients were evaluated together (HHS score 76 out of 100). However, in young female, mainly breast cancer patients with good estimated survival, the scores were > 80, indicating significantly improved results compared with the overall study population. The average OHS was 37 in our study. Based on the classification of Kalairajah et al. (2005), in which a score of 34 to 41 indicates a good functional outcome, a good outcome is represented in our population. In a recent study, Hamilton et al. (2018) defined treatment success following THA based on an OHS threshold of 37.5 points. In our study, one-third of patients had an OHS exceeding this threshold. Again, the best OHSs in our study were seen in young female patients with long survival. Primary tumor and, most importantly, defect defined by Harrington’s classification did not affect the HHS or OHS. In accordance with the literature, our results showed that age strongly affects functional outcome, as the absolute score tends to decrease with age (Bremner-Smith et al. 2004), and a patient’s overall physical status is an important factor in estimating a good functional outcome.

Estimating patient survival is of outmost importance, as reconstruction survival should be longer than patient survival. Since Harrington’s original publication, several modifications of the procedure have been introduced, but the idea of transferring the weight load from the acetabular joint to the intact bone in the ileum over the lytic lesion in the periacetabular area via threaded screws or pins and a reinforcement ring has remained the same. Compared with the literature, our implant survival was superior: 96% at 1, 2, and 5 years, as only 1 reconstruction required revision (Nilsson et al. 2000, Shahid et al. 2014, Tsagozis et al. 2015, Erol et al. 2016, Charles et al. 2017). In the literature, implant survival is rarely reported, but reported implant failure rates leading to revision surgery vary between 3% and 18%. Revisions are needed due to fractured rods, constant or repeated dislocations, and mechanical and technical errors similar to our case (Allan et al. 1995, Marco et al. 2000, Bernthal et al. 2015, Colman et al. 2015). Complications after surgery vary considerably, with 11% in our study and published reports ranging from 16% to 33% (Nilsson et al. 2000, Shahid et al. 2014, Tsagozis et al. 2015, Charles et al. 2017, Krishnan et al. 2017). Complications are often divided into major complications requiring surgery and minor complications, which are treated nonoperatively. Deep infection and recurrent dislocation are common major complications requiring revision surgery. Local tumor progression, in addition to mechanical instability due to insufficient fixation, is more likely to occur in large lesions or long-term survivors (Nilsson et al. 2000, Shahid et al. 2014, Tsagozis et al. 2015, Charles et al. 2017). In accordance with the literature, dislocation and infection were the most common complications in our study, and we did not have any failures due to local tumor growth.

The prognosis of many patients with metastatic bone disease, particularly those without visceral disease, has improved in recent years (Kimura 2018). Although advanced oncological treatment results in the prolonged survival of some patients, almost half of the patients die within the 1st year after surgery for pelvic metastases. Our patient survival rates are in accordance with the literature, with 1-year survival ranging between 42% and 49% (Nilsson et al. 2000, Shahid et al. 2014, Tsagozis et al. 2015). Visceral metastases decrease survival, whereas it is suggested that solitary skeletal metastases, at least in the extremities, be treated with margins to achieve improved survival (Ratasvuori et al. 2014). In our series, the skeletal metastasis was solitary in 18 patients, the surgical margin was always intralesional, and patient survival was similar to patients with more disseminated disease. Increased surgical margins in the periacetabular location result in more massive resection, with an increased rate of complications; therefore, based on our results, we select Harrington’s procedure with intralesional curettage as the method of choice for solitary bone metastases in the periacetabular region.

Our study has several limitations, including those inherent to its retrospective design. The mobility and functional scores were not structurally assessed, and OHS and/or HHS were available for only 53/89 of the patients. In addition, 14/89 of the patients were deceased before the first follow-up and 22/89 were unable to come to a follow-up visit. However, the results can be used to estimate the probable functional outcome in patients with improved survival. A strength of this multi-center study was the large sample size with a homogeneous treatment method for periacetabular metastasis in contrast to previous ones, in which very heterogeneous technical methods were combined. A further strength of the paper was the use of well-validated tools for functional outcome measurement.

In conclusion, functional outcome is mostly dependent on patient-related factors, as young female patients with long survival have good functional outcomes. The results indicate that our modified Harrington’s procedure with the Restoration GAP II ring to achieve stable fixation with bone cement, a constrained acetabular cup to prevent dislocation, and antegrade iliac screws to prevent cranial protrusion is a reliable reconstruction for periacetabular metastases and achieves excellent implant survival in all patients. Though the complication number was 10/89, the reconstruction survival was surprisingly good, at 96% overall. Furthermore, the overall patient survival was 46% at 1 year. Patients with skeletal metastases only had improved survival compared with patients with visceral metastases.

MKL and GK concepted and designed the study. Data extraction was performed by MKL. Data analysis and interpretation were performed by GK and MKL. GK, MN, and MKL were major contributors in writing the article. Article drafting and revising were performed by MKL, JN, VVI, and TKP. The final version was approved by all authors.

**Figure:**
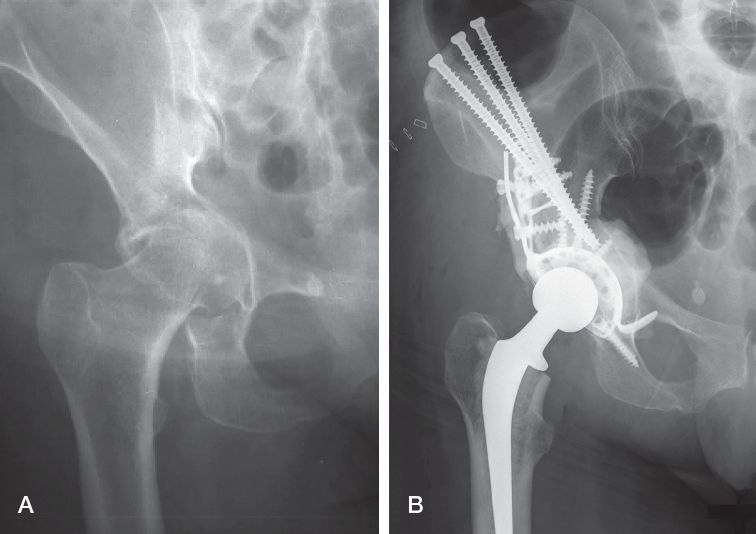
51 year old woman with primary tumor of sigma carcinoma with bone, lung, and liver metastasis. Harrington classification 2 (A). The modified Harrington’s procedure for periacetabular metastases (B). 3 cannulated screws are directed to the roof of the acetabulum and the periacetabular defect is supported by a restoration reinforcement ring. A cemented acetabular cup is implanted. The bone cement is used to augment the bone defects, reinforcement ring, and antegrade inserted screws.
